# Iron metabolism in critically ill patients developing anemia of inflammation: a case control study

**DOI:** 10.1186/s13613-018-0407-5

**Published:** 2018-05-02

**Authors:** Margit Boshuizen, Jan M. Binnekade, Benjamin Nota, Kirsten van de Groep, Olaf L. Cremer, Pieter R. Tuinman, Janneke Horn, Marcus J. Schultz, Robin van Bruggen, Nicole P. Juffermans, Molecular Diagnosis and Risk Stratification of Sepsis (MARS) Consortium, Friso M. de Beer, Lieuwe D. J. Bos, Gerie J. Glas, Roosmarijn T. M. van Hooijdonk, Laura R. A. Schouten, Marleen Straat, Esther Witteveen, Luuk Wieske, Arie J. Hoogendijk, Mischa A. Huson, Brendon P. Scicluna, Tom van der Poll, Lonneke A. van Vught, Maryse A. Wiewel, Marc J. M. Bonten, Jos F. Frencken, Peter M. C. Klein Klouwenberg, Maria E. Koster-Brouwer, David S. Y. Ong, Diana M. Verboom

**Affiliations:** 1 Department of Intensive Care Medicine, Academic Medical Center, University of Amsterdam, Meibergdreef 9, 1105 AZ Amsterdam, The Netherlands; 2 Department of Blood Cell Research, Sanquin Research and Landsteiner Laboratory, Academic Medical Center, University of Amsterdam, Amsterdam, The Netherlands; 3 Department of Research Facilities, Sanquin Research and Landsteiner Laboratory, Academic Medical Center, University of Amsterdam, Amsterdam, The Netherlands; 4 Department of Intensive Care Medicine, University Medical Center Utrecht, Utrecht, The Netherlands; 5 Julius Center for Health Sciences and Primary Care, University Medical Center Utrecht, Utrecht, The Netherlands; 6 Department of Intensive Care Medicine, VU University Medical Center Amsterdam, University of Amsterdam, Amsterdam, The Netherlands

**Keywords:** Critical care, Anemia, Iron, Inflammation, Sepsis, Hepcidin

## Abstract

**Background:**

Anemia occurring as a result of inflammatory processes (anemia of inflammation, AI) has a high prevalence in critically ill patients. Knowledge on changes in iron metabolism during the course of AI is limited, hampering the development of strategies to counteract AI. This case control study aimed to investigate iron metabolism during the development of AI in critically ill patients.

**Methods:**

Iron metabolism in 30 patients who developed AI during ICU stay was compared with 30 septic patients with a high Hb and 30 non-septic patients with a high Hb. Patients were matched on age and sex. Longitudinally collected plasma samples were analyzed for levels of parameters of iron metabolism. A linear mixed model was used to assess the predictive values of the parameters.

**Results:**

In patients with AI, levels of iron, transferrin and transferrin saturation showed an early decrease compared to controls with a high Hb, already prior to the development of anemia. Ferritin, hepcidin and IL-6 levels were increased in AI compared to controls. During AI development, erythroferrone decreased. Differences in iron metabolism between groups were not influenced by APACHE IV score.

**Conclusions:**

The results show that in critically ill patients with AI, iron metabolism is already altered prior to the development of anemia. Levels of iron regulators in AI differ from septic controls with a high Hb, irrespective of disease severity. AI is characterized by high levels of hepcidin, ferritin and IL-6 and low levels of iron, transferrin and erythroferrone.

**Electronic supplementary material:**

The online version of this article (10.1186/s13613-018-0407-5) contains supplementary material, which is available to authorized users.

## Background

Anemia is a hallmark of critical illness, occurring in up to 95% of critically ill patients [1, 2]. The cause of anemia in these patients is often multifactorial including blood loss, low nutrient intake and iatrogenic factors, such as hemodilution and frequent blood sampling. Another major cause of anemia in critically ill patients is anemia of inflammation (AI) [3]. Although distinguishing AI from anemia due to iron deficiency is a diagnostic challenge, the contribution of inflammation to the development of anemia is thought to play a role in up to 75% of critically ill patients [4, 5]. AI is characterized by a decreased production of red blood cells, a shortened red blood cell life span and alterations in iron metabolism, which will impact erythropoiesis [4, 6].

Levels of transferrin, the iron transporter in the circulation, are low in AI, as well as levels of iron [7]. The main regulator of iron levels in the circulation is hepcidin. Hepcidin inhibits iron uptake and transport by internalization of the iron export channel ferroportin on enterocytes, hepatocytes and macrophages, resulting in low levels of iron available for erythropoiesis [8, 9]. Hepcidin production in the liver increases in response to cytokines, such as interleukin 6 (IL-6), whereas both low plasma iron levels and anemia suppress hepcidin. Hepcidin is also regulated by erythroferrone (ERFE), a hormone produced by erythroblasts in response to erythropoietin, which suppresses hepcidin production [10, 11]. During inflammation, cytokines such as IFN-γ inhibit erythropoiesis, resulting in a reduction of the number of erythroblasts [12] and as a result a low ERFE level. Taken together, in AI, due to the high levels of hepcidin, there is not an absolute iron deficiency but rather a decreased iron availability. Consequently, both oral and intravenous supplementation of iron to support erythropoiesis in critically ill patients with anemia has not been unequivocally successful [13–15]. As other means to treat anemia in critically ill, such as supplementation of recombinant erythropoietin, have shown benefit [16], but also harm [6, 17], correction of anemia is usually done by blood transfusions. However, as transfusion is associated with lung injury, infections and increased mortality [2], other strategies to increase iron availability for erythropoiesis are warranted. These may include reducing hepcidin activity, which has been suggested to be beneficial in experimental models [18, 19].

Currently, knowledge on changes in iron metabolism during the course of critical illness is limited, which hampers the development of new strategies to correct AI. In this case control study, we investigated several parameters of iron metabolism in critically ill septic patients who developed anemia during their stay on the ICU. These patients are classified as AI and compared to critically ill control patients with sepsis and without sepsis who have a high hemoglobin level (Hb).

## Methods

### Study design

This is a sub-study of the Molecular Diagnosis and Risk Stratification of Sepsis (MARS) project, which was a prospective observational cohort study on molecular diagnostics of sepsis, conducted in the ICU of 2 tertiary hospitals (ClinicalTrials.gov NCT01905033). All patients admitted to the ICUs between January 2011 and July 2013 older than 18 years and with an expected stay longer than 24 h were included. Trained ICU research physicians prospectively collected demographic data, including Acute Physiology and Chronic Health Evaluation, (APACHE IV score), admission type, daily disease severity scores (Sequential Organ Failure Assessment, SOFA) and outcome. For this study, an opt-out consent method was approved by the Medical Ethical Committees of both centers (IRB No. 10-056C). Participants were informed about the study by a brochure provided at ICU admission attached with an opt-out card that could be completed by the patient or legal representative in case of unwillingness to participate.

### Patient selection

For the current study, three groups of critically ill patients were identified. Patients were classified as developing AI when anemia (Hb < 6 mmol/L) occurred during their ICU stay while complying to the diagnosis of sepsis (termed: AI group). Sepsis was used as criterion for inflammation to be able to identify patients with severe inflammation from the database. Patients with AI were compared to sepsis patients who had a high Hb (Hb level ≥ 7 mmol/L) (termed: septic controls, high Hb) and to patients without sepsis who had a high Hb (termed: non-septic controls, high Hb, *n* = 30 per group) (Additional file 1: Table S1). Hb levels of AI patients and controls were chosen in order to create a clear distinction between patient groups. Anemia was defined as Hb < 6 mmol/L, because these patients near the transfusion trigger and are therefore the clinically relevant anemic patients. The control patients were used to determine the influence of the presence of sepsis on iron metabolism as well as the “background” influence of being critically ill. Sample size of 30 patients per group was chosen based on a previous study that shows statistically significant results with similar patient numbers [20].

For all groups, the following patients were excluded: patients who received red blood cell transfusions prior to or during the inclusion period, patients with conditions which may induce or alter chronic anemia (chronic renal failure, hematological disease, chemotherapy, acquired immunodeficiency syndrome), patients receiving iron or erythropoietin therapy and postoperative patients (in order to avoid patients who became anemic due to blood loss due to invasive procedures). Daily patient files were screened on blood loss, due to surgery or other invasive procedures or gastrointestinal bleeding. Patients with reported blood loss due to invasive procedures or gastrointestinal bleeding directly prior to ICU admission or during the sampling period on the ICU were excluded.

Patients with AI were matched to controls for age and sex using the Optimal Matching method from the MatchIt package of R statistics [21]. Longitudinal blood samples were taken from the biobank of collected samples. Infection was scored and classified using a four point scale (none, possible probable or definite) according to the Center for Disease Control and Prevention [22] and International Sepsis Forum Consensus Conference definitions [23, 24]. Sepsis was defined as a definite or probable infection accompanied by at least one additional parameter as described in the 2001 International Sepsis Definitions Conference [25] (Additional file 2: Table S2). All included sepsis patients had a SOFA ≥ 2 at ICU admission, approximating the new sepsis-3 criteria [26]. To determine whether patients were suffering from iron deficiency, the algorithm of Weiss was used [3].

### Sample selection and analysis

The first blood sampling moment in all groups was on ICU admission. The second sampling moment for AI patients was on the day they developed anemia, the third sampling moment was 2 days later. Control patients were sampled on the first and third day of complying to their classification (Additional file 1: Table S1).

Samples were centrifuged at 1500 g for 15 min at room temperature and plasma was stored at − 80 °C. Measurements were done in heparin anti-coagulated plasma. Serum iron, transferrin, ferritin and haptoglobin were measured by immunoturbidimetric methods (Roche Cobas c702). Transferrin saturation was calculated by the formula serum iron/(25.2 × transferrin). Hepcidin (R&D), soluble transferrin receptor (sTfR) (Biovendor), erythroferrone (MyBioSource) and IL-6 levels (R&D) were measured by enzyme-linked immunosorbent assay kits.

### Statistical analysis

One-way ANOVA was used, or in case of non-normally distributed data, the Kruskal–Wallis. Categorical variables were compared with the Chi-square test or Fisher’s exact tests.

First, it was investigated whether there were differences in iron metabolism between groups. Therefore, the predictive value of the explanatory groups for the iron metabolism variables was assessed. Since the observations over time are nested within patients, analysis was done by a linear mixed model, using the three different groups of ICU patients (AI, septic controls, non-septic controls) as a fixed effect. Patients, which include the repeated measures, were used as random effect. APACHE IV score and proton pump inhibitor (PPI) use were included in the model as potential confounders. The dependent variables in the model showed skewed distributions. The best data transformation from the Box–Cox family of power transformations [27] resulted in log transformations for all iron metabolism variables. The best model fit was investigated with the Linear and Nonlinear Mixed Effects Models package [28]. The modeling process is described in the supplement. Estimates from the log-transformed response scale predictions (levels of iron metabolism variables) were back-transformed and reported. Contrasts among predicted values for groups were tested by the Least-Square Means package [29]. Second, it was investigated whether there were significant differences in levels of iron parameters between sample days using the Friedman test. Mean imputation was used to replace missing data (6 out of 270 data points were missing). Statistical significance was considered to be at *p* = 0.05. All tests were corrected by the Bonferroni method [29, 30]. When appropriate, statistical uncertainty is expressed by the 95% confidence levels. All analyses were performed in R statistics [31].

## Results

Patient selection algorithm is shown in Additional file 3: Fig. S1. Patients in the AI group did not differ from those in the control groups in age and sex, due to matching on these factors (Table 1). Patients with AI, however, tended to be sicker compared to septic and non-septic control groups, exemplified by higher Acute Physiology and Chronic Health Evaluation (APACHE) IV score (*p* = 0.08) and Sequential Organ Failure Assessment (SOFA) scores at admission (*p* = 0.05). However, SOFA scores did not differ between groups in follow-up sampling.

**Table 1:** Patient characteristics

Characteristics	AI (*n* = 30)	Septic controls, high Hb (*n* = 30)	Non-septic controls, high Hb (*n* = 30)	*p* value
Male, *n* (%)	19 (63)	19 (63)	19 (63)	1.00
Age, years median (range)	64.5 (20–85)	65.5 (22–81)	64 (21–79)	0.99
APACHE IV score median (range)	82.5 (45–155)	72 (45–128)	71 (17–104)	0.08
Admission type, *n* (%)				
Surgical ward	7 (23)	4 (13)	7 (23)	0.66
Medical ward	17 (57)	16 (53)	13 (43)	0.75
Neurological ward	6 (20)	10 (33)	10 (33)	0.72
SOFA score median (range)				
Sampling moment 1	7 (3–16)	7 (3–10)	5 (0–15)	0.05
Sampling moment 2	6 (3–17)	6 (1–15)	5 (2–12)	0.09
Sampling moment 3	5 (2–14)	5 (1–15)	4 (1–12)	0.21
Hemoglobin in mmol/L, mean SD				
ICU admission	7.2 (± 0.7)	8.6 (± 0.8)	8.4 (± 0.7)	< 0.01
Second sample	5.6 (± 0.3)	8.1 (± 0.8)	8.1 (± 0.6)	< 0.01
Third sample	5.3 (± 0.3)	8.1 (± 0.7)	8.0 (± 0.6)	< 0.01
ICU mortality, *n* (%)	4 (13)	2 (7)	3 (10)	0.69
Hospital mortality, *n* (%)	10 (33)	6 (20)	9 (30)	0.49

### Hemoglobin level

**Fig. 1:**
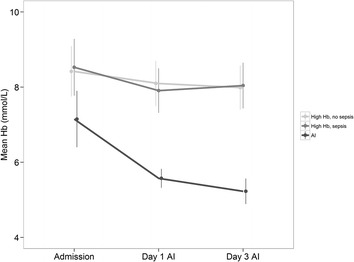
Mean hemoglobin levels over time. Time course of the hemoglobin levels of the three longitudinal sampled groups; anemia of inflammation (AI), septic, high Hb controls and non-septic, high Hb controls. Data are expressed as mean with standard deviation

The course of Hb levels in the different groups is shown in Fig. 1. As per inclusion criteria, the patients in the AI group became anemic over time during the ICU course, but were not anemic when admitted to the ICU. The median time to become anemic in this group was 8 (4–11 interquartile range (IQR)) days. Also per inclusion criterion, the comparative groups (septic controls and non-septic controls) kept a high Hb level during the course of the study. The median sample days for these control groups were similar; admission day 3 (3–3 IQR) and day 5 (5–5 IQR). At admission, the mean Hb levels of the septic and the non-septic control groups were higher than the Hb level of AI patients (*p* < 0.001).

### Iron metabolism in patients with AI

**Fig. 2:**
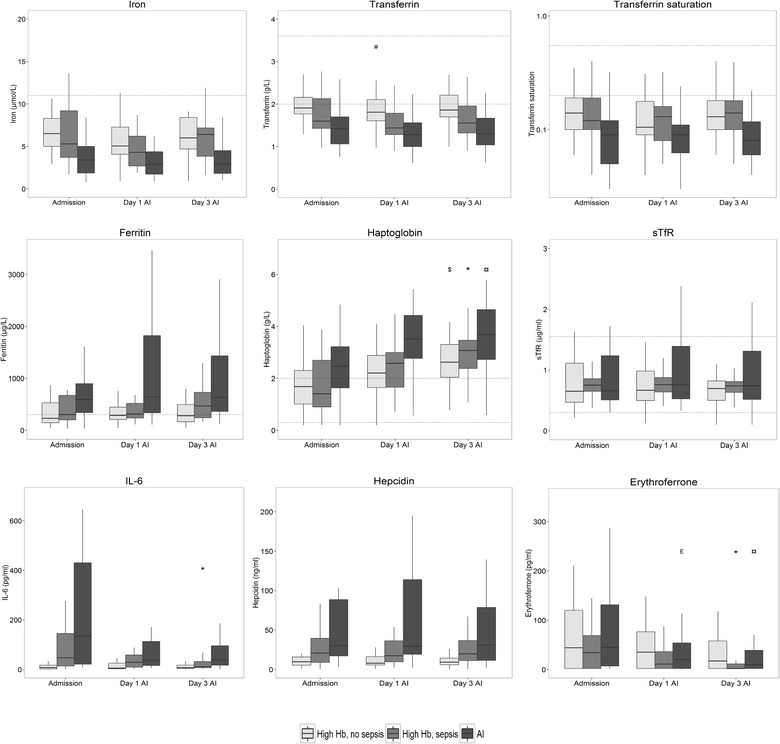
Iron parameters and IL-6 levels per group over time. Time course of observed plasma iron parameters of the three groups; anemia of inflammation (AI), Septic, high Hb controls, non-septic, high Hb controls. Dotted line represents reference values. Statistically significant differences within the groups over time are indicated with: £ *p* < 0.05 AI group ‘Day 1 AI’ compared to ‘Admission’, # *p* < 0.05 non-septic, high Hb group ‘Day 1 AI’ compared to ‘Admission’, $ *p* < 0.05 non-septic, high Hb group ‘Day 3 AI’ compared to ‘Admission’, * *p* < 0.05 Septic, high Hb group ‘Day 3 AI’ compared to ‘Admission’, ¤ *p* < 0.05 AI group ‘Day 3 AI’ compared to ‘Admission’. Data are expressed as median with 25–75 interquartile ranges

In patients in the AI group, the levels of different regulators of iron metabolism were already largely deviating from reference values at ICU admission, even when anemia had not yet developed, and did not change further over time. Iron, transferrin and transferrin saturation were low in AI and did not decrease further over time (Fig. 2). Ferritin levels were increased in AI compared to the reference value and also hepcidin, and IL-6 levels were high, but did not increase further over time (Fig. 2). ERFE levels decreased over time in AI (Fig. 2). Haptoglobin levels were increased at admission compared to reference values and further increased over time (Fig. 2). Taken together, these parameters comply with the diagnosis of AI, characterized by high levels of hepcidin and ferritin and decreased levels of iron and transferrin. Of interest, in AI, iron metabolism was already altered at ICU admission, when Hb levels were still normal.

### Iron metabolism in patients with AI compared to septic and non-septic controls with high Hb level

Table 2 shows the mean estimates derived from the linear mixed model of different parameters of iron metabolism for all patients per group at all time points. Patients in the AI group had a significantly lower iron level compared to septic controls with a high Hb level, as well as a lower transferrin level and a lower transferrin saturation (Table 2). The haptoglobin concentration was significantly higher in AI patients compared to septic controls with a high Hb level. Hepcidin, ferritin, sTfR and ERFE levels were similar. The time course of iron parameters in AI shows a similar pattern as in septic patients with a high Hb level. The haptoglobin level increased over time in both groups and ERFE levels decreased over time, with an earlier decrease in AI compared to septic controls (Fig. 2). This comparison between AI patients and non-septic controls with a high Hb level is similar to the comparison of AI to septic controls with a high Hb level. However, the differences between AI and non-septic controls were more pronounced than the differences between AI and septic controls, suggesting that sepsis influences iron metabolism in AI. However, in the multivariate model, APACHE IV score as a measure of disease severity was not a confounder of results, except for the IL-6 model. Of note, proton pump inhibitors affect intestinal iron intake and are frequently administered to ICU patients [32]. However, the use of proton pump inhibitors was not a confounder of results either (see Additional file 4: Supplement).

**Table 2:** Mean estimates of iron parameters derived from the linear mixed model

Iron parameter	AI	Septic controls, high Hb	Non-septic controls, high Hb
Iron (µmol/L)	3.8 (3.2–4.5)*^†^	5.6 (4.8–6.6)	6.3 (5.4–7.3)
Transferrin (g/L)	1.3 (1.2–1.5)*^†^	1.6 (1.5–1.8)	1.9 (1.7–2)
Transferrin saturation (%)	10 (8–12)*	14 (11–16)	13 (11–15)
Ferritin (µg/L)	1134 (548–2346)^†^	473 (229–976)	314 (152–648)
Haptoglobin (g/L)	3.5 (3.2–3.9)*^†^	2.7 (2.4–3)	2.5 (2.2–2.8)
Hepcidin (pg/ml)	20.7 (15.1–28.5)^†^	12.9 (9.4–17.9)	7.3 (5.4–9.8)
Erythroferrone (pg/ml)	15.5 (9.3–26)	9.6 (5.8–16)	18.7 (11.2–31)
sTfR (µg/ml)	0.83 (0.68–1.01)	0.7 (0.55–0.87)	0.70 (0.57–0.86)
IL-6 (pg/ml)	14.4 (5.1–40.9)^†^	8.0 (1.5–43.0)	2.0 (0.4–10.9)

Data are expressed as (back-transformed) mean estimates of iron parameters for all patients per group at all time points, with 95% confidence interval

Differences between mean estimates are tested by contrasts. * *p* < 0.05 AI compared to septic controls, ^†^ *p* < 0.05 AI compared to non-septic controls

### Contribution of iron deficiency to the development of anemia in patients on the ICU

To determine whether iron deficiency may have contributed to the development of anemia, the algorithm of Weiss [3] was applied to all anemic patients. According to this algorithm, none of the patients with AI were iron deficient.

## Discussion

The current study measured key players of iron metabolism over time in several critically ill patient populations. We show that iron metabolism is altered in ICU patients [20], regardless of the presence of anemia. However, alterations in iron metabolism are more pronounced in AI patients than in non-anemic controls. As patients with AI tended to be more severely ill, we investigated whether these changes could be related to disease severity. The APACHE IV score did not influence outcome of the models, suggesting that not only disease severity drives the development of AI. Hepcidin, which is thought to be the main regulator of AI, was not different between AI and septic controls. ERFE, which regulates hepcidin, showed a rapid decrease in AI patients when compared to septic controls. Therefore, a decrease in ERFE levels may contribute to the development of AI. Haptoglobin levels increased over time in AI, suggesting that intravascular hemolysis does not contribute to the development of anemia during sepsis. However, hemolysis could not be excluded, since increased haptoglobin levels can reflect inflammation, although IL-6 did not increase during AI development. Taken together, iron parameters are altered already at ICU admission. In those patients going on to develop AI, these disturbances are more pronounced compared to septic and non-septic controls, which is not solely due to disease severity.

Our finding that iron metabolism is already altered in AI prior to the development of anemia suggests a window of opportunity to prevent AI. Iron supplementation is an appealing approach. However, in our study, iron deficiency could not be detected in patients with AI. Although the algorithm used to detect iron deficiency has not been validated for ICU patients, AI may account for a large proportion of anemia in ICU patients. Therefore, it may not be surprising that studies on efficacy of iron therapy in critically ill patients showed conflicting results [13–15, 33–35]. Heterogeneity of causes of anemia in patients included in these studies could have contributed to conflicting outcomes. A possible exception is the recent IRONMAN study, which showed that iron supplementation resulted in an increased Hb level at hospital discharge compared to placebo [15]. In this study, patients with a low likelihood of iron deficiency (high ferritin, high transferrin saturation) were excluded, which may have yielded a more homogenous patient population. Given that our study indicates that absolute iron deficiency may not be present in a large proportion of the ICU patients that suffer from anemia, other treatment options may be to increase the amount of iron available for erythropoiesis, e.g. by inhibiting hepcidin activity. An anti-hepcidin antibody reduced the development of AI in monkeys [18] and prevented a fall in iron levels in human endotoxemia [19]. However, the advantages of potential therapies to increase iron availability must be weighed against the risk of adverse events. Iron may promote bacterial growth [36] and increase the risk of infections in several patient populations [37–39]. In ICU patients, high levels of transferrin saturation, which reflects iron availability, has even shown to be a predictor of mortality in septic patients [40]. It is not known whether this association is due to a real pathogenic effect of abundant iron or only reflects a higher katabolic state. Notably, studies on iron therapy in ICU patients did not show an increased rate of in-hospital infections or mortality [13–15, 33, 34].

This study has limitations, the first of which is the small number of patients included. The finding that hepcidin levels were consistently higher in AI compared to septic controls without reaching statistical significance may reflect limited power. Secondly, to define AI patients, sepsis was used as inclusion criterion instead of inflammation, which hampers extrapolation of results to patients with inflammation due to other causes. Thirdly, as a result of the inclusion criteria, iron parameters were not measured at similar timepoints between groups. Finally, patients with liver cirrhosis and occult blood loss were not excluded. Strengths of our study are the case control design and the longitudinal sampling of ICU patients, using control groups which allow to determine the influence of the presence of sepsis on iron metabolism as well as the “background” influence of being critically ill. Also, strict exclusion criteria to limit external factors that influence iron metabolism were applied and strict definitions were used. Further, data and sample collection were performed by dedicated researchers leading to a complete follow-up.

## Conclusions

In conclusion, we have shown that iron metabolism is already changed in AI before anemia occurs, suggesting a window of opportunity for therapy to modulate iron metabolism. Levels of iron, transferrin and transferrin saturation are low in AI patients compared to septic controls, irrespective of disease severity, suggesting that AI is not solely determined by severity of inflammation. ERFE may play a role in the development of AI.

## Additional files

**Additional file 1: Table S1.** Definition of patient groups including sampling days.

**Additional file 2: Table S2.** Diagnostic criteria for sepsis [25].

**Additional file 3: Fig.** **1.** Flowchart of patient selection from database. A total of 8313 patients are included in the MARS database. Of these, 2376 patients stayed at the ICU for ≥ 5 days. 1848 of these patients were admitted to the ICU via a route other than the operating theater. Of these, 1658 did not suffer from chronic kidney failure and were no dialysis patients. Patients were classified as developing AI when Hb ≥ 6 mmol/L at ICU admission and Hb was < 6 mmol/L during their ICU stay for at least 5 days in a row, while complying to the diagnosis of sepsis for at least 3 days in a row. Patients with a Hb ≥ 7 for at least 5 days in a row, with no sepsis scores were classified as ‘High Hb, no sepsis’. Patients with a Hb ≥ 7 for at least 5 days in a row, while complying to the diagnosis of sepsis for ≥ 3 days were classified as ‘High Hb, sepsis’. Patients that received RBC transfusions, EPO or oral iron were excluded, as well as bleeding patients. 46 AI patients, 130 non-septic controls and 89 septic controls were left. Of these, 30 AI patients were matched to controls for age and sex.

**Additional file 4.** Supplement: The modeling process.

## Abbreviations

AI: Anemia of inflammation
APACHE IV score: Acute Physiology and Chronic Health Evaluation IV score
ERFE: Erythroferrone
Hb: Hemoglobin
ICU: Intensive care unit
IL-6: Interleukin 6
IQR: Interquartile range
MARS: Molecular Diagnosis and Risk Stratification of Sepsis
SOFA: Sequential Organ Failure Assessment
sTfR: Soluble transferrin receptor

## References

[1] Corwin HL, Gettinger A, Pearl RG, Fink MP, Levy MM, Abraham E (2004). The CRIT Study: anemia and blood transfusion in the critically ill—current clinical practice in the United States. Crit Care Med.

[2] Napolitano LM, Kurek S, Luchette FA, Anderson GL, Bard MR, Bromberg W (2009). Clinical practice guideline: red blood cell transfusion in adult trauma and critical care. J Trauma.

[3] Weiss G, Goodnough LT (2005). Anemia of chronic disease. N Engl J Med.

[4] Prakash D (2012). Anemia in the ICU: anemia of chronic disease versus anemia of acute illness. Crit Care Clin.

[5] Pieracci FM, Barie PS (2006). Diagnosis and management of iron-related anemias in critical illness. Crit Care Med.

[6] Sihler KC, Napolitano LM (2008). Anemia of inflammation in critically ill patients. J Intensive Care Med.

[7] Ganz T, Nemeth E (2009). Iron sequestration and anemia of inflammation. Semin Hematol.

[8] Krause A, Neitz S, Mägert HJ, Schulz A, Forssmann WG, Schulz-Knappe P (2000). LEAP-1, a novel highly disulfide-bonded human peptide, exhibits antimicrobial activity. FEBS Lett.

[9] Park CH, Valore EV, Waring AJ, Ganz T (2001). Hepcidin, a urinary antimicrobial peptide synthesized in the liver. J Biol Chem.

[10] Kautz L, Jung G, Valore EV, Rivella S, Nemeth E, Ganz T (2014). Identification of erythroferrone as an erythroid regulator of iron metabolism. Nat Genet.

[11] Chen H, Choesang T, Li H, Sun S, Pham P, Bao W (2015). Increased hepcidin in transferrin-treated thalassemic mice correlates with increased liver BMP2 expression and decreased hepatocyte ERK activation. Haematologica.

[12] Libregts SF, Gutiérrez L, De Bruin AM, Wensveen FM, Papadopoulos P, Van Ijcken W (2011). Chronic IFN-γ production in mice induces anemia by reducing erythrocyte life span and inhibiting erythropoiesis through an IRF-1/PU. 1 axis. Blood.

[13] Pieracci FM, Stovall RT, Jaouen B, Rodil M, Cappa A, Burlew CC (2014). A multicenter, randomized clinical trial of IV iron supplementation for anemia of traumatic critical illness. Crit Care Med.

[14] Shah A, Roy NB, McKechnie S, Doree C, Fisher SA, Stanworth SJ (2016). Iron supplementation to treat anaemia in adult critical care patients: a systematic review and meta-analysis. Crit Care.

[15] Litton E, Baker S, Erber WN, Farmer S, Ferrier J, French C (2016). Intravenous iron or placebo for anaemia in intensive care: the IRONMAN multicentre randomized blinded trial. Intensive Care Med.

[16] Corwin HL, Gettinger A, Pearl RG, Fink MP, Levy MM, Shapiro MJ, Corwin MJ, Colton T (2002). Efficacy of recombinant human erythropoietin in critically ill patients a randomized controlled trial. JAMA.

[17] Drueke TB, Locatelli F, Clyne N, Eckardt K, Macdougall IC, Tsakiris D, Burger HU, Scherhag A (2006). Normalization of hemoglobin level in patients with chronic kidney disease and anemia. N Engl J Med.

[18] Schwoebel F, van Eijk LT, Zboralski D, Sell S, Buchner K, Maasch C (2013). The effects of the anti-hepcidin Spiegelmer NOX-H94 on inflammation-induced anemia in cynomolgus monkeys. Blood.

[19] Van Eijk LT, John ASE, Schwoebel F, Summo L, Vauléon S, Zöllner S (2014). Effect of the antihepcidin Spiegelmer lexaptepid on inflammation-induced decrease in serum iron in humans. Blood.

[20] Piagnerelli M, Cotton F, Herpain A, Rapotec A, Chatti R, Gulbis B (2012). Time course of iron metabolism in critically ill patients. Acta Clin Belg.

[21] Ho DE, Imai K, King G, Stuart EA (2011). MatchIt: nonparametric preprocessing for parametric causal inference. J Stat Softw.

[22] Garner JS, Jarvis WR, Emori TG, Horan TC, Hughes JM (1988). CDC definitions for nosocomial infections, 1988. AJIC Am J Infect Control.

[23] Calandra T, Cohen J (2005). The international sepsis forum consensus conference on definitions of infection in the intensive care unit. Crit Care Med.

[24] Klein Klouwenberg PMC, Ong DSY, Bos LDJ, de Beer FM, van Hooijdonk RTM, Huson MA (2013). Interobserver agreement of centers for disease control and prevention criteria for classifying infections in critically ill patients. Crit Care Med.

[25] Levy MM, Fink MP, Marshall JC, Abraham E, Angus D, Cook D (2003). 2001 SCCM/ESICM/ACCP/ATS/SIS international sepsis definitions conference. Crit Care Med.

[26] Singer M (2016). The third international consensus definitions for sepsis and septic shock (sepsis-3). JAMA.

[27] Box GEP, Cox DR (1964). An analysis of transformations. J R Stat Soc Ser B.

[28] Pinheiro J, Bates D, DebRoy S, Sarkar D, R Core Team. nlme: linear and nonlinear mixed effects models. R package version 3.1-128. 2016.

[29] Lenth RV (2016). Least-squares means: the R package lsmeans. J Stat Softw.

[30] Searle SR, Speed FM, Milliken GA (1980). Population marginal means in the linear model: an alternative to least squares means. Am Stat.

[31] RC Team (2016). R: A language and environment for statistical computing.

[32] Vanclooster A, van Deursen C, Jaspers R, Cassiman D, Koek G (2017). Proton pump inhibitors decrease phlebotomy need in HFE hemochromatosis: double-blind randomized placebo-controlled trial. Gastroenterology.

[33] Garrido-Martín P, Nassar-Mansur MI, de la Llana-Ducrós R, Virgos-Aller TM, Rodríguez Fortunez PM, Ávalos-Pinto R (2012). The effect of intravenous and oral iron administration on perioperative anaemia and transfusion requirements in patients undergoing elective cardiac surgery: a randomized clinical trial. Interact CardioVasc Thorac Surg.

[34] Pieracci FM, Henderson P, Rodney JRM, Holena DN, Genisca A, Ip I (2009). Randomized, double-blind, placebo-controlled trial of effects of enteral iron supplementation on anemia and risk of infection during surgical critical illness. Surg Infect (Larchmt).

[35] Madi-Jebara SN, Sleilaty GS, Achouh PE, Yazigi AG, Haddad FA, Hayek GM (2004). Postoperative intravenous iron used alone or in combination with low-dose erythropoietin is not effective for correction of anemia after cardiac surgery. J Cardiothorac Vasc Anesth.

[36] Patruta S, Walter HH (1999). Iron and infection. Kidney Int.

[37] Hoen B (1999). Iron and infection: clinical experience. Am J Kidney Dis.

[38] Sazawal S, Black R, Ramsan M, Chwaya H, Stoltzfus R, Dutta A (2006). Effect of routine prophylactic supplementation with iron and folic acid on admission to hospital and mortality in preschool children in a high malaria transmission setting: community based, randomised, placebo-controlled trial. Lancet.

[39] Brookhart MA, Freburger JK, Ellis AR, Wang L, Winkelmayer WC, Kshirsagar AV (2013). Infection risk with bolus versus maintenance iron supplementation in hemodialysis patients. J Am Soc Nephrol.

[40] Tacke F, Nuraldeen R, Koch A, Strathmann K, Hutschenreuter G, Trautwein C (2016). Iron parameters determine the prognosis of critically ill patients. Crit Care Med.

