# Supplementary material for: Iron metabolism in critically ill patients developing anemia of inflammation: a case control study
Source: Ann Intensive Care. 2018 May 2;8:56. doi: 10.1186/s13613-018-0407-5 (PMC5930297; doi:10.1186/s13613-018-0407-5)
Supplement: Supplementary file 1 — Additional file 1: Table S1. Definition of patient groups including sampling days. [file 13613_2018_407_MOESM1_ESM.doc]

**Table S1.** Definition of patient groups including sampling days.

| **Group** | **Inclusion criteria** | **Sample days** |
| --- | --- | --- |
| **Anemia of inflammation (AI)** | Hb≥6 mmol/L at admission &  Hb<6 mmol/L for ≥ 5 days in a row , of which:  Sepsis score for ≥ 3 days in a row | -Admission  -First and third day Hb<6mmol/L & sepsis score |
| **Septic controls,**  **high Hb** | Hb≥7 mmol/L for ≥ 5 days in a row, of which:  Sepsis score for ≥ 3 days in a row | -Admission  -First* and third day Hb>7mmol/L & sepsis score |
| **Non-septic controls,**  **high Hb** | Hb≥7 mmol/L for ≥ 5 days in a row, of which:  No sepsis scores. | -Admission  -First* and third day Hb>7mmol/L & no sepsis scores |

*When ‘Admission day’ is the same day as ‘First’ day; samples from the first, third and fifth day that patient met inclusion criteria are used for measurement.
