# Supplementary material for: Iron metabolism in critically ill patients developing anemia of inflammation: a case control study
Source: Ann Intensive Care. 2018 May 2;8:56. doi: 10.1186/s13613-018-0407-5 (PMC5930297; doi:10.1186/s13613-018-0407-5)
Supplement: Supplementary file 2 — Additional file 2: Table S2. Diagnostic criteria for sepsis [25]. [file 13613_2018_407_MOESM2_ESM.doc]

**Table S2.** Diagnostic criteria for sepsis [25].

| Infection*,  Documented or suspected and some of the following †: |
| --- |
| General parameters;  -Fever (core temperature >38.3°C)  -Hypothermia (core temperature <36°C)  -Heart rate >90 bpm or >2 SD above the normal value for age  -Tachypnea: >30 bpm  -Altered mental status  -Significant edema or positive fluid balance (>20 ml/kg over 24 h)  -Hyperglycemia (plasma glucose >110 mg/dl or 7.7 mM/l) in the absence of diabetes |
| Inflammatory parameters;  -Leukocytosis (white blood cell count >12,000/µl ^-1^)  -Leukopenia (white blood cell count <4,000/ µl^-1^)  -Normal white blood cell count with >10% immature forms  -Plasma C reactive protein >2 SD above the normal value  -Plasma procalcitonin >2 SD above the normal value |
| Hemodynamic parameters;  -Arterial hypotension † (SBP <90 mm Hg, MAP <70, or an SBP decrease >40 mm Hg in adults or <2 SD below normal for age)  - SO_2_ >70% †  -Cardiac index >3.5 l min^−1^ m^−^2 ‡§ |
| Organ dysfunction parameters;  -Arterial hypoxemia (PaO2/FIO2<300)  -Acute oliguria (urine output <0.5 ml Kg^-1^h^-1^ or 45 mM/l for at least 2h)  -Creatinine increase ≥0.5mg/dl  -Coagulation abnormalities (international normalized ratio >1.5 or activated partial thromboplastin time >60 s)  -Ileus (absent bowel sounds)  -Thrombocytopenia (platelet count 4 mg/dl or 70 mmol/l)  -Hyperbilirubinemia (plasma total bilirubin >4 mg/dl or 70 mmol/l) |
| Tissue perfusion parameters;  -Hyperlactatemia (>1 mmol/l)  -Decreased capillary refill or mottling |

* Defined as a pathological process induced by a micro-organism; † Values above 70% are normal in

children (normally 75–80%) and should therefore not be used as a sign of sepsis in newborns or

children; ‡ Values of 3.5–5.5 are normal in children and should therefore not be used as a sign of

sepsis in newborns or children; § Diagnostic criteria for sepsis in the pediatric population is signs and

symptoms of inflammation plus infection with hyper- or hypothermia (rectal temperature >38.5°C or

<35°C), tachycardia (may be absent in hypothermic patients) and at least one of the following

indications of altered organ function: altered mental status, hypoxemia, elevated serum lactate level,

and bounding pulses.
