# Supplementary figures and images for: Iron metabolism in critically ill patients developing anemia of inflammation: a case control study
Source: Ann Intensive Care. 2018 May 2;8:56. doi: 10.1186/s13613-018-0407-5 (PMC5930297; doi:10.1186/s13613-018-0407-5)

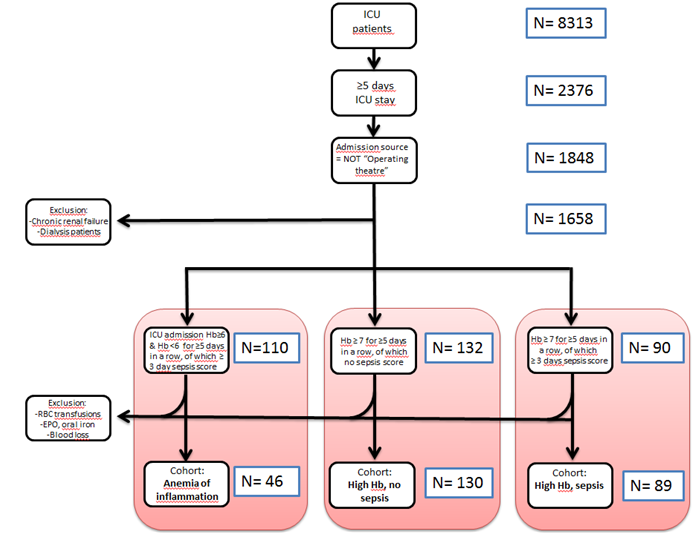

Supplement: Supplementary file 3 — Additional file 3: Fig. 1. Flowchart of patient selection from database. A total of 8313 patients are included in the MARS database. Of these, 2376 patients stayed at the ICU for ≥ 5 days. 1848 of these patients were admitted to the ICU via a route other than the operating theater. Of these, 1658 did not suffer from chronic kidney failure and were no dialysis patients. Patients were classified as developing AI when Hb ≥ 6 mmol/L at ICU admission and Hb was < 6 mmol/L during their ICU stay for at least 5 days in a row, while complying to the diagnosis of sepsis for at least 3 days in a row. Patients with a Hb ≥ 7 for at least 5 days in a row, with no sepsis scores were classified as ‘High Hb, no sepsis’. Patients with a Hb ≥ 7 for at least 5 days in a row, while complying to the diagnosis of sepsis for ≥ 3 days were classified as ‘High Hb, sepsis’. Patients that received RBC transfusions, EPO or oral iron were excluded, as well as bleeding patients. 46 AI patients, 130 non-septic controls and 89 septic controls were left. Of these, 30 AI patients were matched to controls for age and sex. [file 13613_2018_407_MOESM3_ESM.tif]
