# Supplementary material for: Iron metabolism in critically ill patients developing anemia of inflammation: a case control study
Source: Ann Intensive Care. 2018 May 2;8:56. doi: 10.1186/s13613-018-0407-5 (PMC5930297; doi:10.1186/s13613-018-0407-5)
Supplement: Supplementary file 4 — Additional file 4. Supplement: The modeling process. [file 13613_2018_407_MOESM4_ESM.doc]

**Supplement: The modelling process**

This section describes the steps that are taken to find the best model fit, representing the relation between the patient condition groups as explanatory variables and the response variables. Patient condition groups are labeled as: 1] Anemia of inflammation (AI); 2] high Hb, non-septic controls (A-S-); 3] and high Hb, septic controls (A-S+). In this example, hepcidin is used as the response variable. The explanatory variable is the same for all other iron metabolism variables analyzed in this study. In each dataset there are three observations of the response variable per patient with a total of 90 patients.

The response variable (hepcidin) is log transformed to comply to the assumption of normality. It is investigated whether including the APACHE IV score (continuous values) and/or PPI improves the model fit.

First a linear regression model was applied to the data to investigate whether there is a patient effect (repeated measures). The standardized residuals from this model are plotted against patients. The visual inspection of this graph shows that the residuals for each patients are above or below the zero line (figure not shown here). This indicates a ‘within patient correlation’, i.e. dependent observations which violates the assumption of independent observation for a linear regression model. The multiple values of Hepcidin for each patient cannot be regarded as independent from each other.
The next step is to generate a random intercept model, we add a random effect for patient to the model.

*Finding the optimal model*

First, the random intercept model is formulated:

log(hepcidin) = group + Apache4 + PPI + (1|patient) + ε

The log transformed hepcidin are modelled as an intercept (α), plus the group (AI, A-S-, A-S+) effect and an APACHE IV score effect and PPI effect, and all the interactions between these terms, and a random intercept (α_i_) that is assumed to be normally distributed with mean 0 and variance σ_α_^2^, and something that is real noise ε_ij_.

The function Linear Mixed Effects Models (lme) from the R package nlme is used to fit the random intercept model. First, it is assessed whether the mixed effects model is better than the ordinary linear regression model. The gls function without the random intercept was used to refit the linear regression model (R syntax).

M2 <- gls(logwaardeHepcidin ~ group * APACHE_IV_Score * ppi, data = hepcidin)

M3 <- lme(logwaardeHepcidin ~ group * APACHE_IV_Score * ppi,

random = ~1| factor(ID), data = hepcidin)

anova(M2, M3)

AIC’s from both models are compared by the ANOVA test:

Model df AIC BIC logLik Test L.Ratio p-value

M2 1 13 948.7318 994.5627 -461.3659

M3 2 14 871.6333 920.9897 -421.8167 1 vs 2 79.09847 <.0001

The AIC values indicate that the mixed model is preferred. Next, it is investigated whether the random intercept improves by adding a random slope to the model. The group and APACHE IV score and PPI effects are subsequently included as random slopes, because they can be different per patient. The difference between the models is evaluated again by the ANOVA test.

The random intercept model appeared to be the one with the best fit.

Model df AIC BIC logLik Test L.Ratio p-value

M2 1 13 948.7318 994.5627 -461.3659

M3 2 14 871.6333 920.9897 -421.8167 1 vs 2 79.09847 <.0001

M4 3 16 869.7674 926.1747 -418.8837 2 vs 3 5.86586 0.0532

M4b 4 16 872.3897 928.7970 -420.1949

M5 5 19 872.8622 939.8458 -417.4311 4 vs 5 5.52753 0.1370

Extending the models with different random slopes (M4 and M5) gives no improvement in model fit (M3). Therefore the random intercept model is still the best model. Visually inspection of the plotted residuals of the M3 model showed a random distribution (not shown here).

The current model (M3) includes the interaction terms between groups and APACHE IV score and PPI. Do we still need those, i.e. do they improve the model? The full model (M3) was compared with sub models for testing the contribution of Apache IV score and PPI.

We tested the model with and without PPI as interaction term. Being negative, we tested the contribution of PPI as main effect, which also showed no contribution to the model fit. The same was done for Apache IV score which was also removed from the model showing not to contribute to the mode fit (AIC criteria, p value < 0.05).

*The final model*

Mfinal <- lme(logwaardeHepcidin ~ group, random = ~1| factor(patient),

data = Hepcidin, method = "ML")

From the output of the model summary, the model can present with values for the explanatory variable

log(hepcidin) = 10.58 + -1.53*A-S- + -0.76*A-S+ + α_i_ + ε_ij_

α_i_ ~ N(0, 0.98^2^). for the within patient residuals we have ε_ij_ ~ N(0, 0.86^2^)

- If an observation is labeled by AI, then the expected Hepcidin

log(hepcidin) = 10.58 + -1.53*0(A-S-) + -0.76*0 (A-S+) + α_i_ + ε_ij_

- If an observation is labeled by A-S-, then the expected Hepcidin

log(hepcidin) = 10.58 + -1.53*1(A-S-) + -0.76*0 (A-S+) + α_i_ + ε_ij_

- If an observation is labeled by A-S-, then the expected Hepcidin

log(hepcidin) = 10.58 + -1.53*0(A-S-) + -0.76*1 (A-S+) + α_i_ + ε_ij_

To develop the best model for each tested iron parameter in this study, the same steps as describe here were completed. APACHE IV score and PPI did not contribute to these models.

Summary model parameter estimates

| **Table.** Model parameter estimates (Beta, 95% CI and *p* value) for the relation between the log transformed response variables and the categorical explanatory variable. | | | |
| --- | --- | --- | --- |
|  | **Model parameters: Beta (95% CI), *p* value** | | |
| **Log transformed response variables** | **Reference (AI)** | **Septic controls,**  **high Hb** | **Non-septic controls, high Hb** |
| Iron (µmol/L) | 1.09 (0.93 to 1.26),  *p* <0.0001 | 0.52 (0.28 to 0.75),  *p* <0.0001 | 0.59 (0.36 to 0.83),  *p* <0.0001 |
| Transferrin (g/L) | 0.28 (0.18 to 0.38),  *p* <0.0001 | 0.17 (0.04 to 0.31),  p = 0.014 | 0.33 (0.19 to 0.46),  *p* <0.0001 |
| Transferrin saturation (%) | -0.21 (-0.42 to -0.01),  *p* = 0.037 | -0.16 (-0.47 to 0.15),  *p* = 0.298 | -0.17 (-0.46 to 0.12),  *p* = 0.247 |
| Ferritin (µg/L) | 6.65 (6.26 to 7.05),  *p* <0.0001 | -0.67 (-1.23 to -0.11),  *p* = 0.019 | -1.01 (-1.56 to -0.45),  *p* = 0.001 |
| Haptoglobin (g/L) | 1.04 (0.86 to 1.22),  *p* <0.0001 | -0.32 (-0.58 to -0.07),  *p* = 0.013 | -0.41 (-0.66 to -0.15),  *p* = 0.002 |
| Hepcidin (pg/ml) | 10.58 (10.18 to 10.97), *p* = <0.0001 | -1.53 (-2.1 to -0.97),  *p* = <0.0001 | -0.76 (-1.33 to 0.2),  *p* = 0.009 |
| Erythroferrone (pg/ml) | 2.71 (2.19 to 3.23),  *p* = <0.0001 | -0.47 (-1.21 to 0.27),  *p* = 0.213 | 0.2 (-0.54 to 0.94),  *p* = 0.596 |
| sTfR (µg/ml) | -0.21 (-0.42 to -0.01),  *p* = 0.037 | -0.16 (-0.47 to 0.15),  *p* = 0.298 | -0.17 (-0.46 to 0.12),  *p* = 0.247 |
|  | | | |
